# Evaluation of Glycemic Index Education in People Living with Type 2 Diabetes: Participant Satisfaction, Knowledge Uptake, and Application

**DOI:** 10.3390/nu12082416

**Published:** 2020-08-12

**Authors:** Shannan M. Grant, Andrea J. Glenn, Thomas M. S. Wolever, Robert G. Josse, Deborah L. O’Connor, Alexandra Thompson, Rebecca D. Noseworthy, Maxine Seider, Melissa Sobie, Gurita Bhatti, Julianne Cavanagh, Emily Jones, Pauline B. Darling

**Affiliations:** 1 Department of Applied Human Nutrition, Mount Saint Vincent University, 166 Bedford Highway, Halifax, NS B3M 2J6, Canada; 2 Department of Nutritional Sciences, University of Toronto, 1 King’s College Circle, Toronto, ON M5S 1A8, Canada; andrea.glenn@utoronto.ca (A.J.G.); thomas.wolever@utoronto.ca (T.M.S.W.); robert.josse@unityhealth.to (R.G.J.); deborah.oconnor@utoronto.ca (D.L.O.); Alex@breastfeedingcollective.com (A.T.); rebecca.noseworthy@sickkids.ca (R.D.N.); maxineseider@gmail.com (M.S.); pdarling@uottawa.ca (P.B.D.); 3 Clinical Nutrition and Risk Factor Modification Centre, St. Michael’s Hospital, 61 Queen Street E, Toronto, ON M5C 2T2, Canada; 4 Nutrition Department, St Michael’s Hospital, 61 queen St. E, Toronto, ON M5B 1W8, Canada; melissa.sobie@sunnybrook.ca (M.S.); gurita.bhatti@gmail.com (G.B.); julianne.cavanagh@unityhealth.to (J.C.); enjones@osmh.on.ca (E.J.); 5 Division of Endocrinology and Metabolism, St. Michael’s Hospital, 61 Queen St. E, Toronto, ON M5C 2T2, Canada; 6 Physiology and Experimental Medicine Program, Hospital for Sick Children, 555 University Avenue, Toronto, ON M5G 1X8, Canada; 7 School of Nutrition Sciences, Faculty of Health Sciences, University of Ottawa, Ottawa, ON K1N 7K4, Canada

**Keywords:** glycemic index OR glycaemic index, diabetes, education evaluation, Kirkpatrick Model, nutrition education, integrative knowledge translation strategy, behavior change, practice-based research, patient-focused intervention

## Abstract

The glycemic index (GI) has been included in the Canadian clinical practice guidelines for type 2 diabetes (T2D) management since 2003, and even longer in other parts of the world (e.g., Australia). Despite this, dietitians have reported that GI is “too difficult for patients to understand and apply.” They have called for diverse GI-utility data and evidence-informed education materials. To address these concerns, we developed and evaluated a GI education workshop and supporting materials, using the Kirkpatrick Model, for a T2D population. Participants (*n* = 29) with T2D attended a dietitian-facilitated workshop and received education materials. A mixed-form questionnaire (GIQ) and 3-day-diet-record were used to capture patient demographics, satisfaction, knowledge, and application, prior to and immediately after the workshop, 1-week, and 4-weeks post-education. Dietary GI was significantly lower at 1 and 4 weeks post-education (mean ± SEM; both 54 ± 1), compared to pre-education (58 ± 1; *p* ≤ 0.001). Participants (28/29) were satisfied with the intervention. The GI knowledge score was significantly higher post-education at baseline (83.5 ± 3.4%; *p* ≤ 0.001), week one (87.5 ± 2.6%; *p* = 0.035), and week four (87.6 ± 3.8%; *p* = 0.011) when compared to pre-education (53.6 ± 5.1%). A significant reduction in dietary GI was achieved by participants living with T2D, after completing the workshop, and they were able to acquire and apply GI knowledge in a relatively short period.

## 1. Introduction

Diabetes Canada Clinical Practice Guidelines (CPG, 2012–2018) include recommendations that glycemic index (GI) education be used as part of medical nutrition therapy (MNT) for patients living with type 1 (T1D) and type 2 diabetes (T2D) (T1D: Grade B, Level 2; T2D: Grade B, Level 2) [1,2]. Other global diabetes clinical practice guidelines include comparable recommendations (e.g., Australia) [3,4,5,6,7,8]. This said, 61% of Canadian Registered Dietitians (RDs) working with patients with diabetes mellitus (DM) do not use GI in practice [9,10]. These data are representative of GI use in other health professions, engaging in nutrition education for DM [11,12]. The following four barriers to GI utility have been identified by educators: (1) lack of suitable GI education tools, (2) a belief that GI is too difficult for patients to understand and (3) too difficult to apply, and (4) a desire for additional GI utility data from diverse patient populations [11]. In support of the first barrier, a Canadian-based evaluation of GI education materials, using the Suitability Assessment of Materials (SAM) instrument (by Doak et al. 1996 [13]), conducted in 2011, showed that available GI education materials are “unsuitable” (SAM rating) [14]. There was, however, insufficient evidence to support or refute claims that GI is too difficult for patients to understand and apply [12,15,16,17,18,19]. This study is one example of end-of-grant dissemination and is part of a larger integrative knowledge translation (iKT) initiative developed and implemented to address all four barriers. Moreover, although many systematic reviews and meta-analyses and clinical trials have evaluated the effect of a low GI dietary intervention on biochemical and/or anthropometric outcomes, the majority of these studies do not comprehensively evaluate GI education or use pedagogical theory/practices [20,21,22].

**Figure 1:**
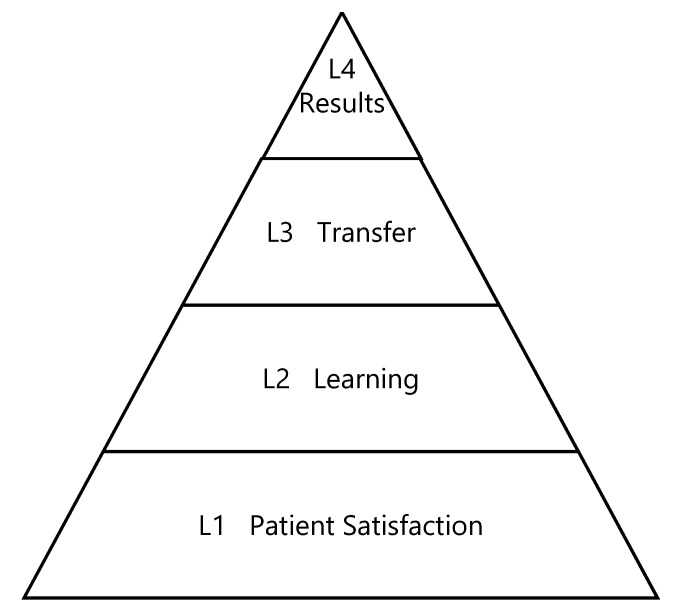
Kirkpatrick method—the four levels. Adapted from Kirkpatrick Partners, LLC. All rights reserved. Reproduced, with permission. Visit http://www.kirkpatrickpartners.com for more information.

Dietary intervention complexity is well recognized and examined in the literature [23,24,25,26,27]. Bonell et al. [28] and others recognize that dietary interventions cannot be effectively evaluated in absence of educational and behavioral outcomes [24,26,29,30,31,32]. According to the Kirkpatrick Model (KM) [33] and other education/training development and evaluation frameworks (e.g., Miller’s Pyramid, Kern’s Six Step Guide to Curriculum Design [34,35]), comprehensive education-based intervention evaluation requires more than traditional clinical measures of utility (e.g., glycemic control) [33,34,35]. The GI Education Evaluation Study (GIEES) was developed to address this gap and educator-identified barriers. The GI education tools and approaches implemented in GIEES represent an evidence-based, patient-focused education platform and intervention evaluation strategy. Classical KM was used as the theoretical framework for intervention evaluation [33], as it is an evidence-based approach to training and education evaluation, adapted for use in health care settings [36]. KM includes the following four levels: (1) Level 1 (L1): Reactions (e.g., patient satisfaction), (2) Level 2 (L2): Learning (e.g., knowledge uptake and knowledge score), (3) Level 3 (L3): Transfer (e.g., behavior change and change in dietary GI), and (4) Level 4 (L4): Results (e.g., glycemic control and improved β-cell function) (Figure 1). According to KM, to achieve L4, one must ensure the bottom three levels are satisfied first. Evaluation of the effect of GI education has traditionally focused on examining biochemical and/or anthropometric outcomes (KM L4 or “results”) [20,21,22]. Therefore, this study has focused evaluation on the bottom three levels (L1–3) (Figure 1). The primary objective of this study was to evaluate if a low GI education platform can significantly reduce dietary GI in participants with T2D. Our hypothesis was that patients living with T2D, who participate in a GI education session, will be satisfied (KM L1) with the session, show an increase in GI knowledge (KM L2), and significantly lowered their dietary GI post-education in comparison to presession dietary GI (KM L3). The overall goal of this study was to develop (and test) an education and evaluation framework that could be used in subsequent trials and by other researchers and clinicians.

Level 1 (Patient Satisfaction): To what degree do patients react positively to the education? Level 2 (Learning): To what degree do patients increase their knowledge, based on their participation in the education session? Level 3 (Transfer): To what degree do patients apply what they learned in their daily lives (i.e., do they change their behavior)? Level 4 (Results): To what degree do favorable outcomes occur as a result of education?

## 2. Materials and Methods

### 2.1. Design and Research Ethics Review

The GIEES was a pre- and post-education evaluation design with repeated postintervention measures; immediately after education, at 1-week and 4-week post-education. The questionnaire (Glycemic Index Questionnaire or GIQ) and GI Education Platform (workshop + patient and trainer education materials) were developed (face- and content-validated and pretested according to methodology outlined by Peterson 2000 [37]).

### 2.2. Participants

Study participants were recruited from St Michael’s Hospital Diabetes Comprehensive Care Program (DCCP) from March to September, 2012. Individuals meeting four criteria were eligible for GIEES participation: (1) diagnosed with T2D, (2) receiving care within St Michael’s Hospital DCCP, (3) over the age of 18, and (4) able to speak and read English. Original ethics review was obtained by St. Michael’s Hospital Research Ethics Board (REB) and University of Toronto Office of Research Ethics. Secondary data analysis was provided by Mount Saint Vincent University Research Office and REB (for dissemination). All study participants provided their written informed consent. The sample size calculation showed that 26 participants would be required to detect a 5-unit difference in the primary outcome (dietary GI) at 80% power [38]. The standard deviation of the difference (in GI) seen by Burani and Longo (2006) [38] was 8.7 units. A drop out of 10% was estimated, resulting in a final sample size of 29 participants.

### 2.3. Intervention

The intervention included a 45-min GI education workshop, facilitated by a trained registered dietitian and two dietitian students/trainees (G.B., M.S., J.C., and E.J.). The workshop occurred six times to accommodate participants’ schedules and to facilitate small group sizes (*n* = 4–10). A PowerPoint presentation was used to guide the interactive workshop and to layer GI education onto standard care. Two evidence-based education materials were provided to participants: (1) The Low Glycemic Index Food Substitution List (recently published as the revised Diabetes Canada Glycemic Index Food Guide [33] and (2) The Low Glycemic Index Recipe Booklet (recently published by Dietitians of Canada Learning on Demand [39]). The workshop concluded with an innovative interactive patient-led game designed to combine standard care and glycemic index knowledge and skills based on Diabetes Canada’s Plate Method and available via Dietitians of Canada Learning on Demand.

Using food substitution lists to guide participant intake is called the “key foods strategy”; recognized as effective in achieving moderate and measurable modifications in food intake [12,15]. Participants were instructed to substitute as many carbohydrate containing foods that they could, but, were assured that one substitution per meal was enough to lower dietary GI. Previous studies have found that a significant difference in dietary GI (5–9 units) can been achieved using this approach, which can replace 60% of starchy foods (in a diet composed of 45–65% carbohydrate) with lower GI carbohydrates [15,40,41,42]. The GIEES iteration of the food substitution list included foods from all Canadian Food Guide food groups (2007) [10,43,44,45]. The practice of including all food groups in GI education is an approach that is commonly used in Australian GI education (Brand-Miller and colleagues) [46]. This practice was adopted to provide participants with more options and to communicate the importance of layering GI education onto current recommendations and, therefore, addressing the concern held by some that promoting a low GI diet may result in patients choosing ice cream (GI = 25 to 80 units, depending on type) or chocolate (GI = 25 to 87 units, depending on type) more often [47]. In addition, the GIEES food substitution list used the traffic light method [12]. This categorical color-based method is used to distinguish between three GI categories: (1) Green = Low GI = Go = Choose Most Often), (2) Yellow/amber = Medium GI = Caution = Choose Less Often, and (3) Red = High GI = Stop and think = Choose Least Often. This method for distinguishing GI categories was made popular by Slabber (2005) and has been used internationally in GI education/nutrition communication [12,48,49].

### 2.4. Data Collection

Date of T2D diagnosis was collected from participants’ medical charts. Informed by the KM hybrid evaluation template and peer-reviewed questionnaires/surveys, the GIQ was developed to evaluate the intervention [33]. The GIQ is a face/content validated, mixed-format (open- and closed-end) questionnaire with four sections, designed to evaluate GI education and based on the first three levels of KM (Tables S1–S4 in Supplementary Materials include GIQ questions and response options). The four sections of the GIQ are: (1) Section 1 (Participant Satisfaction), (2) Section 2 (Demographic Information), (3) Section 3 (GI Knowledge), and (4) Section 4 (Acceptability and Application of GI Education). Baseline administration was completed at the Risk Factor Modification Centre (RFMC) at St. Michael’s Hospital (with research team support), while subsequent section administrations were completed at home by participants (with telephone-based support) and mailed to the RFMC. The GIQ was administered with a 3-day diet record at baseline, postintervention at the baseline visit, 1-week postintervention, and 4-weeks postintervention.

### 2.5. Statistical Analysis

#### 2.5.1. Quantitative Analysis

A GIQ Data Entry Handbook was developed in 2012, during protocol development, to standardize GIQ data entry and analysis, based on methods described by Taylor-Powell and Renner and others [50,51,52]. Data, obtained via closed-ended questions, were entered directly into IBM SPSS version 21 (SPSS Inc., Chicago, IL, USA) (Copyright IBM Corporation and other(s) 1989, 2012) from GIQ, for descriptive and inferential statistical analysis. 

Date of T2D diagnosis was used to calculate years with condition and presented as a mean ± SD and used for descriptive purposes. GIQ Sections 1–4 were subjected to descriptive analysis and presented as counts and/or percentages. Within group comparisons between repeated measures (“visits”) were completed for GIQ Sections 3 and 4. Comparisons for GIQ Section 3 GI knowledge (mean score ± SEM at each visit) were conducted by applying a paired *t*-test, in the context of the close-testing procedure. GIQ Section 4 data (collected 1- and 4-weeks post-education) were analyzed using the Wilson Score Method (confidence intervals (CIs) or binomial proportion). Data collected using the Likert scale were collapsed into dichotomous values (e.g., >good = 1, <good = 0; Likert scale). Dietary intake was analyzed using ESHA Research’s Food Processor SQL: Nutrition Analysis and Fitness Program (Copyright 2012, ESHA Research, Salem, OR, USA). Dietary intake data was analyzed using linear mixed models, to assess change in dietary glycemic index and other dietary variables over the study period. Specifically, dietary intake data was analyzed using a fixed effects model (intercept and visit) and maximum likelihood (ML). Models were adjusted for baseline intake and the Sidak correction was applied for multiple comparisons. All statistical tests were given a 2-tailed *p* < 0.05 criterion of significance, and all data analyses were conducted using Microsoft Excel 2013 (Microsoft Redmond, Washington, DC, USA) or IBM SPSS version 21 (Copyright IBM Corporation and other(s) 1989, 2012).

#### 2.5.2. Qualitative Analysis

Open-ended GIQ responses were reviewed by two reviewers (dietitians) who coded these data according to categories/themes. A data table and theme codebook were developed to further prepare these data for sorting and analysis; with guidance from the aforementioned GIQ Data Entry Handbook. The two reviewers worked independently, in duplicate, keeping detailed summary notes for each theme/code identified. The reviewers then came together to compare themes, codes, and notes to compare interpretations and conclusions; consensus was obtained.

## 3. Results

### 3.1. Participant Characteristics

Thirty-nine DCCP patients signed the letter of information, 30 patients consented to participate and 29 attended and completed the baseline visit. One participant withdrew half-way through the baseline visit because of time limitations (due to work demands). Participants completed the study if they completed the baseline appointment (in full) and either week 1 and/or week 4 post-education. Demographic and clinical characteristics of the participants are shown in Table 1. Overall, participants were mainly identified as male (69%), were of European descent (62%), were highly educated (55% with undergraduate degree or above), and treated their diabetes with oral medications (51%). Only 17% of participants had received previous education on the glycemic index from a healthcare professional, although the majority of participants (90%) had met with a dietitian to discuss diet prior to joining GIEES.

#### 3.1.1. Satisfaction—Kirkpatrick Model L1

The closed-end responses to the education were universally positive; the options disagree and strongly disagree were not selected. Closed-end questions asked participants about their perceptions of their learning (e.g., did you learn anything new? Did the handouts help you understand?), experience (e.g., likes and dislikes), and perception of intervention effectiveness (e.g., I think that what I learned today will help me make changes to my diet.). All participants reported that reviewing standard care before GI education was very helpful. They reported that the GI concept reinforced standard care, despite the fact that 90% of study participants had seen a dietitian in the past and received standard care MNT (Table 1). Twenty-eight participants (97%) responded “yes” to question 2 (“Did you learn anything new at this GI class?”). Twenty-nine participants (100%) provided written responses/qualitative data to question three, “If you learned something today, what did you learn?” The most notable themes established by reviewers were: (1) Current Standard Care: Serving Size (6 responses) (2) GI: Participant Perception of GI Knowledge (14 responses), and (3) GI: GI Categories (11 responses).

The first theme (Current Standard Care) was developed to capture instances where standard care recommendations (i.e., serving size and food groups) were reinforced by the GI education. Three examples of participant responses (*n* = 3) that were organized under subtheme “serving size” include: (1) “Quantity—especially my carrots—although (they are) good, (I know) don’t overdo it,” (2) “The most important thing is that I know how much food I should eat and what kind of food,” (3) “Pasta, rice are not bad choices! Milk is a good choice. (important to remember) portion control.” Other relevant subthemes identified in these quotes include: “Perception of Food/Diet Quality” (e.g., “milk is a good choice”) and “Perception of Dietary Liberation” (e.g., “pasta and rice are not bad choices”). Participants consistently reported that they had “more dietary choices” and felt “less restricted” postworkshop contradicting a common criticism that MNT or “diets” are restrictive [53,54].

The second theme (GI) was developed to capture data specific to GI education and relevant KM levels (L1–L3). GI subthemes “Participant Perception of GI Knowledge” and “GI Categories” were the most commonly observed during analyses, with 14 and 11 statements/responses being identified, respectively. Participants perceived that they learned new information and provided both qualitative (above) and quantitative (L2) evidence. Increased comprehension and application of knowledge, related to the aforementioned GI categories (traffic light) and food substitutions, stood out during qualitative analysis. Four examples of statements falling under relevant subthemes (e.g., Categories, Comprehension) are: (1) “Which breads are low(er) glycemic… how to lower the GI of a meal,” (2) “Low vs. Medium vs. High GI foods and how to (use this information to) manage food and blood glucose levels,” (3) “Food (the same one) can have different GI based on how it is cooked and served,” (4) “Better understanding of GI as it apples (applies) to carb-fruit-veg section of the CFG (Eating Well with Canada’s Food Guide 2007) [43].”

Eleven participants (38%) responded to question five, “What can we do to make the class better?” Responses to this question called for development of a theme called “Feedback from Participants.” Four subthemes were established under this theme, including: (1) scheduling/timing of class, (2) class flow/message cohesion, (3) class content/information, and (4) education delivery strategies (e.g., props, games, and interaction). These data were used to inform subsequent versions of the workshop and implemented in subsequent clinical trials [55,56]. Perhaps the most noteworthy feedback were comments that highlighted utility of the game. For instance, one participant said, “The class moved along well and was made interesting with the use of food products (models) to help build a food plate. Visual goes a long was in driving home the point of what it takes to achieve blood sugar at acceptable levels.”

#### 3.1.2. Knowledge Uptake—Kirkpatrick Model L2

**Figure 2:**
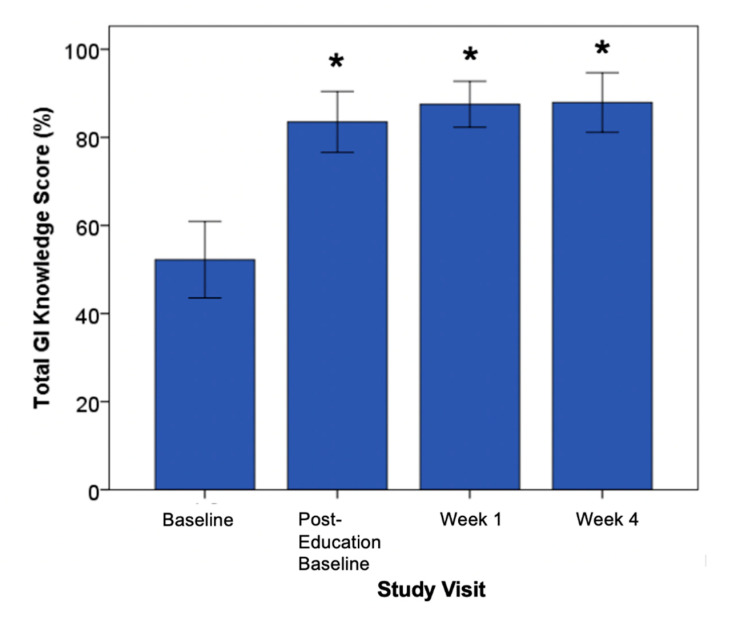
Glycemic index knowledge score at each study visit (Glycemic Index Questionnaire (GIQ) Section 3). * Significantly different from baseline score; mean (± SEM); *p* < 0.05.

Study participants scored significantly higher on the knowledge section of the GIQ after the GI education. The GI knowledge score was significantly higher post-education at baseline (83.5 ± 3.4%; *p* ≤ 0.001), week 1 (87.5 ± 2.6%; *p* = 0.035), and week 4 (87.6 ± 3.8%; *p* = 0.011) (Figure 2) when compared to pre-education (53.6 ± 5.1%) (Figure 1). There were no significant differences between GI knowledge scores post-education (post-education baseline, week 1 post-education, and week 4 post-education). This knowledge score was maintained until the end of the study, indicating sustained knowledge uptake.

#### 3.1.3. Behavior Change/Transfer—Kirkpatrick L3

The primary outcome, dietary GI (a direct measure of behavior change), was significantly lower at week 1 and week 4 (54 ± 1 at each visit) when compared to baseline dietary GI (58 ± 1) (*p* ≤ 0.001). Dietary fiber and protein did not change from baseline during the post-education study period. Calories, carbohydrate, and fat were significantly lower postintervention, with no significant difference between week 1 and week 4. Macronutrient intake, as a percentage of total daily caloric intake(s), was not significantly different between visits/weeks and was maintained within the acceptable macronutrient distribution ranges (AMDR) [57] (Table 2).

GIQ Section 4 was designed to measure participants’ perception of their own skill/application, self-efficacy, time and monetary burden, and participants’ perception of the effect of their diet change on others in their life. This section also measured participant intention to continue with the low GI diet poststudy, and participants’ acceptability of the food included in the food list. The responses to the GIQ on applicability and acceptability of low GI foods were remarkably positive and were supportive of improved adherence (Table 3). By week one, 75% of respondents reported adding low GI foods to their diet and by week four, 88% did. By week one, 79% of respondents reported being good, very good, or excellent (≥good) at choosing low GI foods in the supermarket. Moreover, 70% of respondents rated their ability to make traditional meals with low GI foods as good, very good, or excellent. Furthermore, 100% of participants indicated they would continue to consume low GI foods after the study at the final visit (week 4).

## 4. Discussion

Participants receiving the dietitian-facilitated GIEES education platform were satisfied with the education (KM L1), showed an increase in GI knowledge (KM L2), and demonstrated decreased dietary GI (KM L3). Most importantly, the results of the GIQ did not support the statement “GI is too hard for patients to understand and apply” (perceived barriers from the literature) [9,10]. We found the opposite: participants were able to increase their GI knowledge score and reduce their dietary GI post-education, in a relatively short period of time, without experiencing barriers commonly reported by participants engaging in “healthy eating” or dietary change (e.g., too expensive, hard to find in grocery store, and contradictory to traditional eating patterns) [10]. For instance, we found, by 4 weeks post-education, 75% of respondents reported low GI foods did not take more time to prepare and did not cost more. This is in line with previous research conducted by our Australian colleagues, where they showed that women were able to follow low GI dietary advice during pregnancy with no significant increase in daily costs [58]. Cost of diet is an outcome of extreme relevance to clinicians at St. Michael’s Hospital, as it serves a diverse inner-city patient population, including patients without housing, recent immigrants/refugees, and lower income families.

Another common criticism of the low GI diet is that it opposes current dietary guidelines (e.g., the diet can be used to justify high fat, high sugar choices) [10]. This criticism has not been supported by the literature and is further disputed by our results [10]. For instance, macronutrient intake, as a percentage of total daily caloric intake, was not significantly different between week 1 and 4 post-education. However, the intervention brought carbohydrate and fat (as a percentage of daily total energy) within the AMDR, while protein was within the AMDR from baseline to end of the study. Moreover, energy, dietary carbohydrate, and fat intake (in grams/day) decreased post-education. We did not determine if the decreased energy intake was related to the healthy eating education or low GI education (or both) or resulted in weight loss, in this one-armed study, however, a recent systematic review and meta-analysis has shown that low GI diets can result in weight loss in patients with diabetes compared to a variety of isocaloric control diets [22]. Fiber intake was maintained throughout the study (not statistically different between visits), indicating the dietary intervention successfully controlled for fiber intake which was an aim of the intervention. The mean change in dietary GI was four GI units; a decrease that resulted in a dietary reclassification from medium GI to low GI (< 55), which is indicative of behavior change (KM L3) and successful GI education application. It is also in line with current Diabetes Canada Clinical Practice Guidelines (2018) for Prevention and Management of Diabetes (Type 1, Type 2, and gestational diabetes) [2] and other international diabetes clinical practice guidelines [3,4,5,6,7,59].

The pre-education score (~54%) was lower than expected by many participants; 83% of participants reported hearing about GI before joining the study and 66% reported “knowing what GI is.” This was not surprising, however, as 83% of study participants reported they had never been taught about GI by a healthcare professional before [60,61]. This is despite 90% of participants reporting that they had met with a dietitian to talk about diet, before joining GIEES. These findings are supportive of the existing literature on dietitians use of GI in DM management [3]. In fact, our findings suggest that GI may be used less by dietitians in the DCCP in comparison to other Canadian RDs [9,62]. Our results show that participants’ knowledge score increased postintervention and was maintained during the study period. Increased GI knowledge, accompanied by increased consumption of low GI foods, was also seen by authors of a recent study that found men with more GI knowledge reported lower dietary GI [15,62]. These data are also in agreement with work we have previously published [15,36,41].

A decrease in 5 to 9 GI units in dietary GI, achieved in our study, has been associated with improvements in T2D treatment outcomes (e.g., glycemic control and weight management) [15,40,41,42,45,63,64]. This decrease in dietary GI has been achieved with substitution of 60% of carbohydrate foods in the diet and is a direct measure of behavior change. Previous work by our group also showed that we were able to facilitate a 9 unit decrease in dietary GI in women living with GDM, with resulting improvements in glycemic control [15]. The baseline dietary GI of the GIEES participants was 58 GI units, which is identical to the pre-education dietary GI observed in our previous and current work [15,56,65]. This may also support the participants’ perception that they may already be consuming a lower GI diet (they were consuming a diet on the lower end of the medium GI category). This is illustrated by one participants’ statement related to his/her experience with adding low GI foods to his/her diet: “I was already eating mostly low GI foods, so there is not a great change.”

### 4.1. Strengths and Limitations

A unique strength of this work was the rigor exerted to ensure evidence-based approaches for education and evaluation development and evaluation were applied and members of the research team were trained with Kirkpatrick Partners (S.M.G.).

This study does, however, have several limitations that were kept in mind when interpreting the results. The study sample was representative of the patient population served by St Michael’s Hospital DCCP and the larger Toronto population, in that approximately 50% of our participants were born in another country and immigrated to Canada [66,67]. That said, the sample had a relatively high number of people identifying as European, compared to the larger Toronto population and other Toronto-based research, which can be considered as a limitation. The achievement of a statistically significant increase in knowledge and behavior change during our study may be related to the level of education reported by the participants. Over 85% of respondents reported completing a college certificate or diploma, undergraduate degree, or graduate degree. This likely facilitated knowledge uptake, comprehension, and ability to consume low GI foods. This is in agreement with a previous study of highly educated participants (63% had at least some college or university education), where the researchers found that higher income (likely reflective of higher education) resulted in higher intentions to consume a low GI diet [68]. It is important to consider, from a pedogeological perspective, however, that prior learning and capacity for critical thinking and feedback exchange was a key component of GIQ development/pretesting and GI education evaluation and is in agreement with key methodology and tenets of KM and iKT [15,69]. Moreover, there are ongoing efforts to evaluate if lower income/less educated populations would perceive our low GI education platform similarly. This is an aim of active research on GI being conducted by our group in Atlantic Canada, an area of Canada often highlighted for its high poverty rates with high incidence and prevalence of chronic disease (including diabetes) and home to three of our authors (S.M.G., A.J.G., and R.D.N.) [70,71]. Another limitation related to the knowledge change during our study is that the repeated testing of the GIQ may have facilitated knowledge retention over the study period. Future studies will explore different approaches of education testing (e.g., baseline and end of study only) to address this limitation.

This study was also conducted before the new version of Canada’s Food Guide (2019) [72] was released, and therefore, the workshop content was layered onto Eating Well with Canada’s Food Guide (2007) [43]. The new Canada’s Food Guide applies the plate method, which is a key component of Diabetes Canada education materials and was the stimulus for the game we included in the workshop. Moreover, Diabetes Canada, like many groups providing nutrition education support to medicalized populations, currently continues to use food groups. The GI education materials were layered onto Diabetes Canada education materials (e.g., the plate method) and have been developed to be versatile. That is, they can be layered onto a variety of medical nutrition therapy approaches. Our education has also been integrated into an active clinical trial using the new Canada’s Food Guide [56]. 

There is the potential that many may perceive the shorter study period of 4 weeks as a limitation. Some GIEES participants reported “feeling” that they did not have enough time to make dietary changes; illustrated by the following participant quotes: “…not enough time has passed to be indicative of substantive change or not,” and “This process takes time to incorporate low GI recipes and to grocery shop for new ingredients, e.g., still on same loaf of white bread (as at baseline).” This may be a potential reason for the relatively small decrease in dietary GI observed in our participants, which was short compared to other published GI studies [15,16,40,42]. Nevertheless, it is arguably more notable that our participants were still able to obtain a significant decrease in dietary GI, despite the short time. This contradicts perceptions that the low GI diet is too difficult to understand and apply and highlights that a low GI intervention is relevant to patient populations where behavior change is made in a short period of time (i.e., gestational diabetes) [9,73].

### 4.2. Implications for Practice and Research

This study showed that layering GI education on top of established T2D standard care (e.g., the plate method) resulted in a low GI dietary pattern, in agreement with dietary reference intakes and showed potential for energy reduction. It also showed that a dietitian-facilitated glycemic index intervention can result in satisfaction and knowledge uptake. Although statistical correlations between KM level(s) were not explored, it is well established in the pedogeological literature that participant satisfaction, knowledge, and behavior change all impact dietary intervention effectiveness [33]. Subsequent work has collected qualitative and quantitative data on Registered Dietitian’s and Certified Diabetes Educator’s GI education self-efficacy and examined dietary intake data comprehensively (e.g., including sugars) [65]. Most importantly (from our perspective), our/these findings provide an educational and evaluation framework for future clinical trials (e.g., NCT01589757 and NCT04272840). Moreover, subsequent research in diverse patient populations, applying more recent adaptations of the KM (New World Kirkpatrick Model, including four levels and more), is justified and ongoing [74]. For instance, we are in the process of writing a manuscript on SAM analysis of the Canadian GI education materials, available through Diabetes Canada and Dietitians Canada, and for which, this work is foundational.

## 5. Conclusions

In conclusion, people living with T2D can successfully lower dietary GI after using an evidence-based GI education platform, informed by the first three levels of KM (satisfaction, knowledge uptake, and behavior change). Our findings support previous peer-reviewed literature that reports that a low GI diet is a satisfactory and sustainable dietary approach (medical nutrition therapy) for T2D prevention and treatment.

## Figures and Tables

**Table 1:** Participant demographic and clinical characteristics (Glycemic Index Questionnaire (GIQ) Section 2).

Characteristic, *n* (%)	All Participants (*n* = 29)
Time since diabetes diagnosis, mean years ± SD	7 ± 8
Sex, female	9 (31)
Ethnicity	
European descent	18 (62)
Aboriginal	2 (7)
African/Caribbean	1 (3)
West Indian	1 (3)
South Asian	2 (7)
East Asian	2 (7)
Southeast Asian	2 (7)
Mixed	1 (3)
Born in Canada	15 (52)
English main language spoken at home	24 (83)
Who purchases food most often?	
I do	20 (69)
Spouse/partner	5 (17)
Housekeeper	1 (3)
Who cooks meals most often?	
I do	19 (66)
Spouse/partner	7 (24)
Children	1 (3)
Housekeeper	1 (3)
Highest level of education received	
Less than high school	2 (7)
High school or equivalent	2 (7)
College certificate or diploma	10 (34)
Undergraduate degree or higher	16 (55)
How diabetes is treated	
Lifestyle only	3 (10)
Oral medications	15 (51)
Oral medications + insulin	9 (31)
Insulin	2 (7)
Met with a dietitian before to discuss diet	26 (90)
Heard of the glycemic index before	24 (83)
Know what the glycemic index is	19 (66)
Been taught about the glycemic index from a health care professional before	5 (17)

SD = standard deviation.

**Table 2:** Participant energy and nutrient intake data at baseline and week 1 and 4 post-education from the 3-day diet records.

Dietary Intake Outcome, Mean ± SEM	Baseline	Week 1	Week 4
Calories (kcal)	1965 ± 67	1647 ± 73 *	1631 ± 74 *
Carbohydrate, total (g)	222 ± 8	183 ± 9 *	187 ± 9 *
Fiber (g)	21 ± 1	22 ± 1	23 ± 1
Protein (g)	95 ± 4	86 ± 4	91 ± 4
Fat (g)	76 ± 4	63 ± 4 *	58 ± 4 *
Glycemic Index (%)	58 ± 1	54 ± 1 *	54 ± 1 *
Carbohydrate, total (% of total energy), mean	44	45	44
Fat (% of total energy), mean	36	35	34
Protein (% of total energy), mean	20	20	22

* Significantly different from baseline; *p* < 0.05. SEM = standard error of mean.

**Table 3:** Application and acceptability of the glycemic index education postintervention (GIQ Section 4).

GIQ Question	Week 1, *n* (%)	Week 4, *n* (%)
I have added low GI foods to my diet since week 1	18 (75)	21 (87)
Percentage of total intake has been made up of low GI foods		
<51%	15 (63)	13 (54)
≥51%	9 (37)	11 (46)
Your ability to choose low GI foods in the supermarket		
Good or higher	19 (79)	17 (71)
Fair or less	5 (21)	7 (29)
Your ability to choose low GI foods when eating outside of the home		
Good or higher	13 (54)	16 (63)
Fair or less	11 (46)	9 (37)
Your ability to include low GI foods in meal planning		
Good or higher	16 (67)	18 (75)
Fair or less	8 (33)	6 (25)
Your ability to make traditional meals with low GI foods		
Good or higher	15 (65)	18 (75)
Fair or less	8 (35)	6 (25)
The people you live with been eating low GI foods since week 1		
Yes	8 (44)	10 (67)
No	10 (56) ^a^	5 (33) ^b^
How would your house mates rate the low GI foods?		
Good or higher	11 (58)	4 (25)
Fair or less	8 (42) ^c^	12 (75) ^d^
Planning meals with low GI foods does not require more time	16 (70)	18 (75)
Low GI foods cost the same as other foods	17 (74)	18 (75)
I will continue to eat low GI foods after the study is over	23 (96)	24 (100)

GI = glycemic index; GIQ = Glycemic Index Questionnaire, week 1 and week 4 are both post-education ^a^ Six participants selected “this question does not apply to me”; remaining percent expressed out of 18 participants. ^b^ Nine participants selected “this question does not apply to me”; remaining percent expressed out of 15 participants ^c^ Five participants selected “this question does not apply to me”; remaining percent expressed out of 19 participants ^d^ Seven participants selected “this question does not apply to me”; remaining percent expressed out of 16 participants.

## Supplementary Materials

The following are available online at https://www.mdpi.com/2072-6643/12/8/2416/s1, Table S1: Questions from Glycemic Index Questionnaire (GIQ) Section 1: How did you like the glycemic index (GI) class?; Table S2: Questions from GIQ Section 2: Getting to know you; Table S3: Questions from GIQ Section 3: What do you know about GI?; and Table S4: Questions from GIQ Section 4: Is your low GI diet working for you?
