# Supplementary material for: Evaluation of Glycemic Index Education in People Living with Type 2 Diabetes: Participant Satisfaction, Knowledge Uptake, and Application
Source: Nutrients. 2020 Aug 12;12(8):2416. doi: 10.3390/nu12082416 (PMC7469042; doi:10.3390/nu12082416)
Supplement: Supplementary file 1 [file nutrients-12-02416-s001.pdf]

Supplementary Materials

Table S1: Questions from GIQ Section 1: How did you like the GI class?

**Open-ended questions**
If you learned something new today, what was the most important thing you learned?
What can we do to make the class better?
**Close-ended questions**
Who are you?<br>▪ Patient / friend of patient / spouse of partner of patient
Did you learn anything new at this GI class?<br>▪ Yes / no
The class content was easy to understand<br>▪ Strongly agree / agree / neither agree nor disagree / disagree / strongly disagree
The class content was interesting<br>▪ Strongly agree / agree / neither agree nor disagree / disagree / strongly disagree
The teacher was easy to understand<br>▪ Strongly agree / agree / neither agree nor disagree / disagree / strongly disagree
The teacher's assistants were easy to understand<br>▪ Strongly agree / agree / neither agree nor disagree / disagree / strongly disagree
The handouts helped me learn the class content<br>▪ Strongly agree / agree / neither agree nor disagree / disagree / strongly disagree
The presentation helped me learn the class content<br>▪ Strongly agree / agree / neither agree nor disagree / disagree / strongly disagree
The hands-on activities helped me learn the class content<br>▪ Strongly agree / agree / neither agree nor disagree / disagree / strongly disagree
I think that what I learned today will help me make changes to my diet

▪ Strongly agree / agree / neither agree nor disagree / disagree / strongly disagree

Table S2: Questions from GIQ Section 2: Getting to know you

**Open-ended questions**
If you were not born in Canada, what year did you move here?
What is the main language you speak at home?
Please list all jobs you have held in the past year for more than one month
**Close-ended questions**
What is your sex?<br>▪ Male / female / pass
Which ethnic group do you identify with?<br>▪ Aboriginal / European / African or Caribbean / West Indian / Latin American / Indian / Middle Eastern / East Asian / South East Asian / other
Were you born in Canada?<br>▪ Yes / no
In your home, who purchases the food most often?<br>▪ I do / my spouse or partner / my parent(s) / my children / my roommate / other
In your home, who makes the meals most often?<br>▪ I do / my spouse or partner / my parent(s) / my children / my roommate / other
What is the highest level of education you have finished?<br>▪ High school or equivalent / college certificate or diploma / undergraduate degree / graduate degree / other
How do you treat or control your diabetes today? Choose all that apply.<br>▪ Diet / exercise / oral medication / insulin / other
Have you ever met with a dietitian before to talk about diet?<br>▪ Yes / no / I am not sure
Have you ever heard of the glycemic index?<br>▪ Yes / no / I am not sure
Do you know what the glycemic index is?<br>▪ Yes / no

Have you been taught about the glycemic index from a health professional before?<br>▪ Yes / no

Table S3: Questions from GIQ Section 3: What do you know about GI?

**Close-ended questions**
Which of the words below best describes your knowledge of GI?<br>▪ Poor / fair / good / very good / excellent
The GI is a tool we can use to group carbohydrate foods by the effect they have on blood sugar<br>▪ True / false / I do not know
Which breakfast food has the lowest GI?<br>▪ Corn flakes / white bread / bran buds / oatmeal / bran flakes / I do not know
Which meal has the lowest GI?<br>▪ Canned pasta in tomato sauce / "al dente" spaghetti in tomato sauce / instant white rice with tomato sauce / a hamburger on a white bun with French fries / I do not know
The GI is a number between 1 and 100<br>▪ True / false / I do not know
According to the Canadian Diabetes Association, a high GI food is a food that has a GI of:<br>▪ Less than 55 / between 56 and 69 / 70 or more / I do not know
The GI of a white potato will be the lowest when served as:<br>▪ A cold potato salad with olive oil / a hot baked potato with butter / a warm boiled potato with salsa / a warm potato salad with olive oil / I do not know
Which snack food has the highest GI?<br>▪ Popcorn / Ryvita rye crisps / pretzels / couscous / I do not know
Which foods are absorbed most quickly?<br>▪ Low GI foods / medium GI foods / high GI foods / acidic foods / I do not know
Foods that are broken down fast by the body are:<br>▪ Medium GI foods / high GI foods / low GI foods / super foods / I do not know

The GI ranks food by how much it raises<br>▪ Flatulence (farting) / blood protein / calories / blood sugar / I do not know
Medium to high GI foods will raise your blood sugar ____ than low GI foods<br>▪ Faster and higher / slower and higher / slower and lower / medium to high GI foods will not affect your blood sugar / I do not know
Fresh meat, fish and poultry have a GI of:<br>▪ 0 / meat, fish and poultry do not have a GI / 55 / between 72 and 100 / I do not know

Table S4: Questions from GIQ Section 4: Is your low GI diet working for you?

**Open-ended questions**
How would you describe your experience adding low GI foods to your diet?
**Close-ended questions**
Since the GI class, I have added low GI foods to my diet<br>▪ True / false
Since the GI class, what percentage of your total intake of starchy foods has been low GI?<br>▪ 0% / 0 to 25% / 26 to 50% / 51 to 75% / 76 to 100% / I am not sure
Please choose the word below that best describes your ability to choose low GI foods in the supermarket<br>▪ Poor / fair / good / very good / excellent
How would you rate your skill at choosing low GI foods when eating out of the home?<br>▪ Poor / fair / good / very good / excellent
How would you rate your skill at using low GI foods in meal planning?<br>▪ Poor / fair / good / very good / excellent
How would you rate your ability to make traditional meals with low GI foods?<br>▪ Poor / fair / good / very good / excellent / this question does not apply to me
Since the class, have the people you live with been eating low GI foods?<br>▪ Yes / no / this question does not apply to me
Overall, how would you say your house mates would rate the low GI foods?

- Poor / fair / good / very good / excellent / this question does not apply to me

Have you noticed any changes in the symptoms, listed below, since the GI class?

- Constipation / hunger between meals / bloating / diarrhea / nausea / stomach ache / gas or flatulence
    - Answer yes / no and increase / decrease for each

How would you rate the taste of the lower GI food choices you have made since the GI class?

- Poor / fair / good / very good / excellent

Planning low GI meals does not require more time than planning other meals

- True / false

Low GI foods cost the same as other foods

- True / false

Do you think you will continue to eat low GI foods after this study is over?

- Yes / no
